# An Unusual Presentation of Third-Degree Atrioventricular Block in a Patient With Hypertrophic Cardiomyopathy

**DOI:** 10.7759/cureus.89041

**Published:** 2025-07-30

**Authors:** Emmanuel Gbee, Moses Kiwanuka Ssebuliba, Doreen Nakagaayi

**Affiliations:** 1 Cardiology, Uganda Heart Institute, Kampala, UGA; 2 Internal Medicine, John F. Kennedy Medical Center, Monrovia, LBR; 3 Adult Cardiology, Uganda Heart Institute, Kampala, UGA

**Keywords:** hypertrophic cardiomyopathy, implantable cardioverter defibrillator, sudden cardiac death, syncope, complete av dissociation

## Abstract

Patients with hypertrophic cardiomyopathy (HCM) are commonly affected by ventricular tachyarrhythmias such as ventricular tachycardia, leading to syncope and sudden cardiac death (SCD). Complete atrioventricular (AV) block in patients with HCM is very unusual but may also lead to syncope and SCD. We report a 52-year-old male who presented with recurrent episodes of pre-syncope and effort intolerance. A 12-lead ECG demonstrated deep T-wave inversion in the precordial leads with complete AV dissociation, and a two-dimensional echocardiogram revealed HCM without resting or provoked left ventricular outflow tract obstruction. The patient initially got a temporary transvenous pacemaker, followed by a dual-chamber rate-responsive pacemaker, which was subsequently upgraded to a dual-chamber implantable cardioverter-defibrillator after further risk stratification. Although rare, there have been a few reported cases of HCM complicated by atrioventricular block. This case should alert physicians to the possibility of atrioventricular block in patients with HCM, which could influence the management outcomes.

## Introduction

Hypertrophic cardiomyopathy (HCM) is a common genetic disorder affecting 0.2-0.5% of the general population [1]. Syncope or pre-syncope occurs in approximately 25% of patients with HCM [2].

The incidence of HCM in Uganda is not known but there have been sporadic cases seen at the Uganda Heart Institute, though none have presented with complete atrioventricular (AV) dissociation. However, in other parts of Africa, as reported by Falase et al., there were 0.2% of 6680 unselected echocardiograms of HCM cases in Tanzania and 2% of 712 echocardiograms in Lagos, Nigeria. In Ghana, 1.15% of 572 patients referred for echocardiography at the Ghana National Cardiac Reference Center had HCM, while Abegaz in Ethiopia discovered that 53 out of 1240 abnormal echocardiograms performed at the Armed Forces General Hospital had HCM [3]. No reports of atrioventricular dissociation were mentioned in those cases.

Recurrent syncope is known as one of the risk factors for sudden cardiac death (SCD), and while arrhythmias are common in HCM and may lead to syncope and SCD, bradyarrhythmia or conduction disturbance, though uncommon, may also be a cause of syncope or sudden death. Understanding the mechanism causing loss of consciousness is important as it guides proper treatment and prevents SCD.

However, the incidence of arrhythmias in HCM is well documented. In a review by the Pediatric Electrophysiology Society of 135 children and adolescents with HCM, routine ECG showed arrhythmias in 39% of patients. These arrhythmias include atrial fibrillation/flutter, supraventricular tachycardia, atrioventricular conduction disease, sinus node disease, and ventricular arrhythmias [4].

In this review, we report a 52-year-old male who was diagnosed with HCM and presented with recurrent pre-syncope and complete heart block (CHB). He subsequently had a dual-chamber implantable cardioverter defibrillator (ICD). To the best of our knowledge, this should be the first case of such at the Uganda Heart Institute and probably in Uganda.

## Case presentation

A 52-year-old male who had been apparently well prior to his illness presented to our Coronary Care Unit with a year history of recurrent episodes of lightheadedness, dizziness, and near collapse. His symptoms were mainly on exertion and had been steadily worsening, interfering with his activities of daily living, which made him to seek care at our facility after attempts at other facilities to no avail. He had no history of any medical illness, use of cigarettes, alcohol, or other illicit substances. He reports that two of his siblings died in their mid-thirties and forties, but the actual causes of death were not known; however, from the description, they were not sudden and seemed to be long-standing illnesses. There was no other reported family history of cardiovascular diseases.

**Figure 1:**
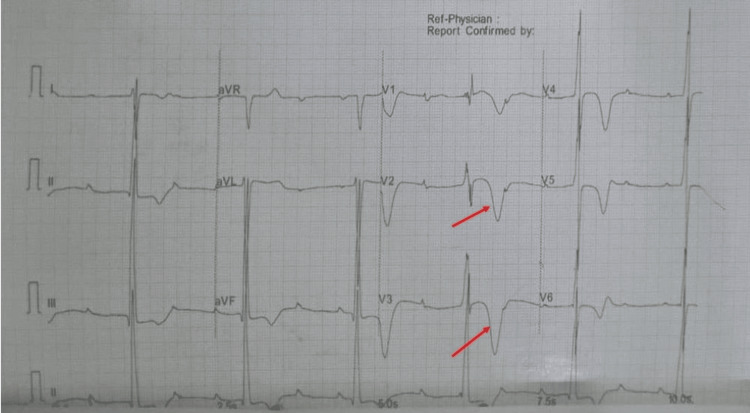
A 12-lead ECG showing a complete AV dissociation and the deep T-wave inversion in V1-V5 (red arrows). AV: atrioventricular.

On examination, he was in a fair general condition, afebrile with no pallor, jaundice, edema, or rashes. His cardiovascular exam was significant for bradycardia (35 bpm) and a mildly elevated blood pressure of 148/85 mmHg. Apex beat was in the fifth interspace with heart sounds I and II, and S4 was heard. Other systems were all unremarkable. The baseline ECG showed CHB, with a heart rate of 32 bpm, a ventricular escape rhythm, and deep T-wave inversion in the precordial and inferior leads, more marked in the precordial leads (Figure 1).

**Figure 2:**
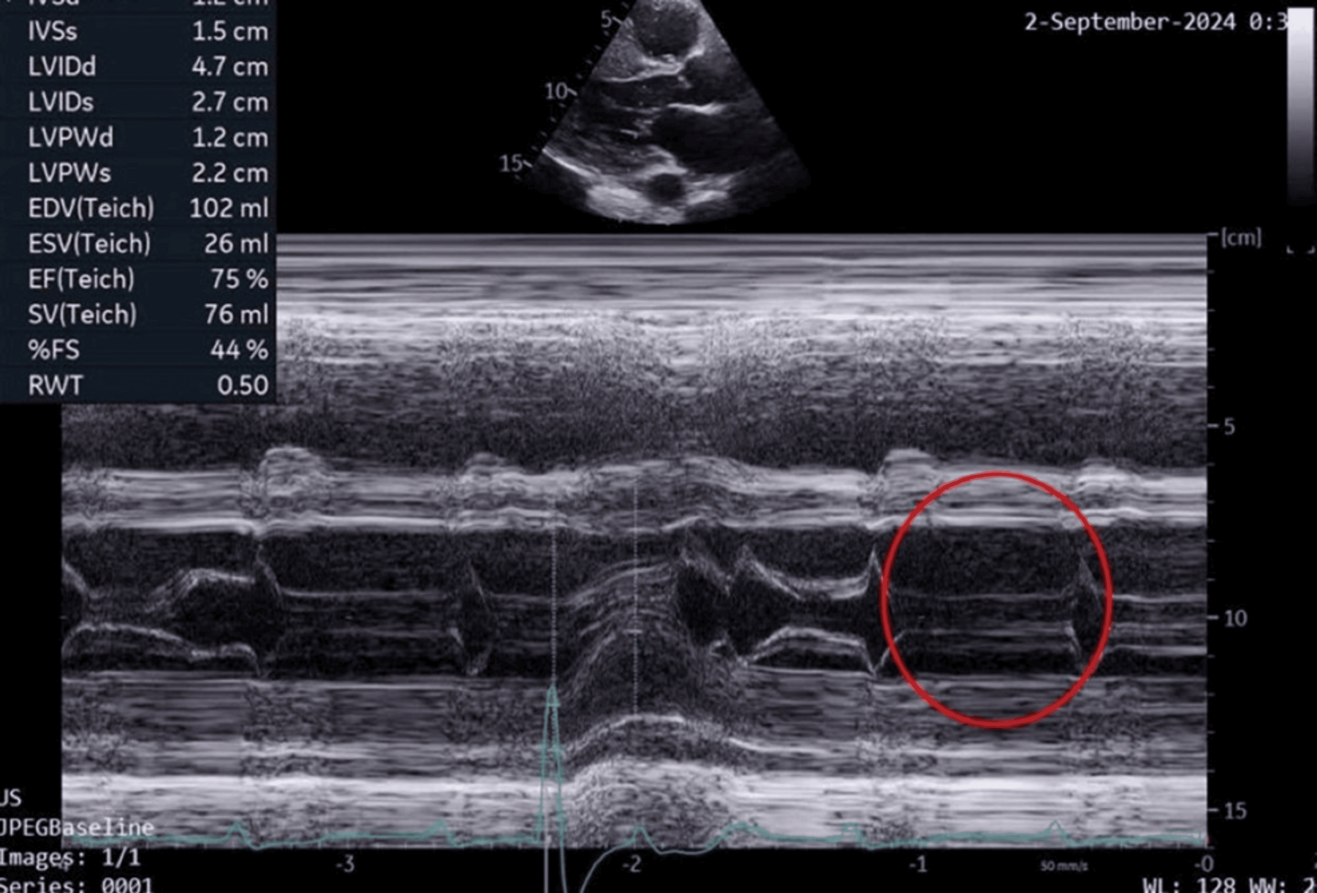
M-mode showing no features of systolic anterior mitral leaflet movement (red circle).

**Figure 3:**
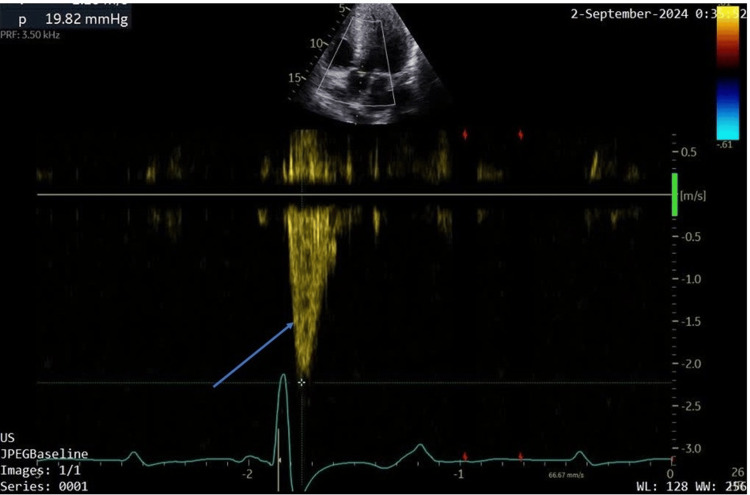
Continuous wave Doppler showing no significant gradient across the left ventricular outflow tract (blue arrow).

**Figure 4:**
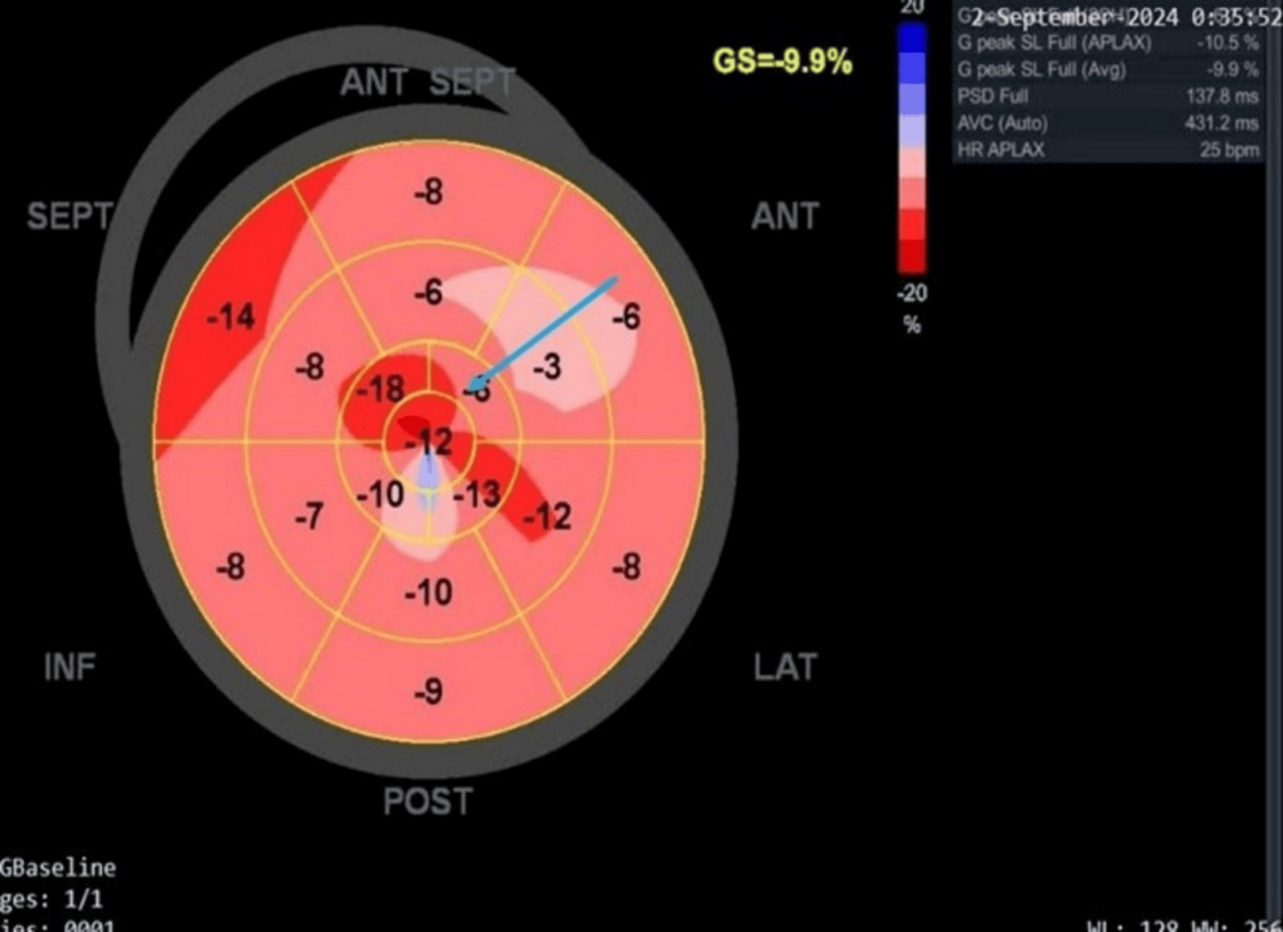
The Bull's eye showing reduced global longitudinal strain (blue arrow).

A two-dimensional echocardiogram (Videos 1, 2) revealed asymmetric hypertrophy with marked mid-septal and apical hypertrophy of 30 mm and 31 mm, respectively. There was no resting or provoked left ventricular outflow tract obstruction (LVOT) or mid-cavity gradients, and no systolic anterior motion (SAM) of the anterior mitral valve (as seen in Figure 2 of the M-mode). The left atrium was mildly dilated (41 mm) (Figure 3), but cavity dimensions and systolic function (75%) of the left ventricle were within normal limits. Speckle tracking revealed an average global longitudinal strain of 9.9% (Figure 4), which was significantly reduced with areas of reduced contractility consistent with areas of hypertrophy on imaging.

**Video 1:** Parasternal long-axis view showing the hypertrophied left ventricle, mainly the septal wall.

**Video 2:** Apical four-chamber view showing the septal and apical left ventricular wall hypertrophy.

**Figure 5:**
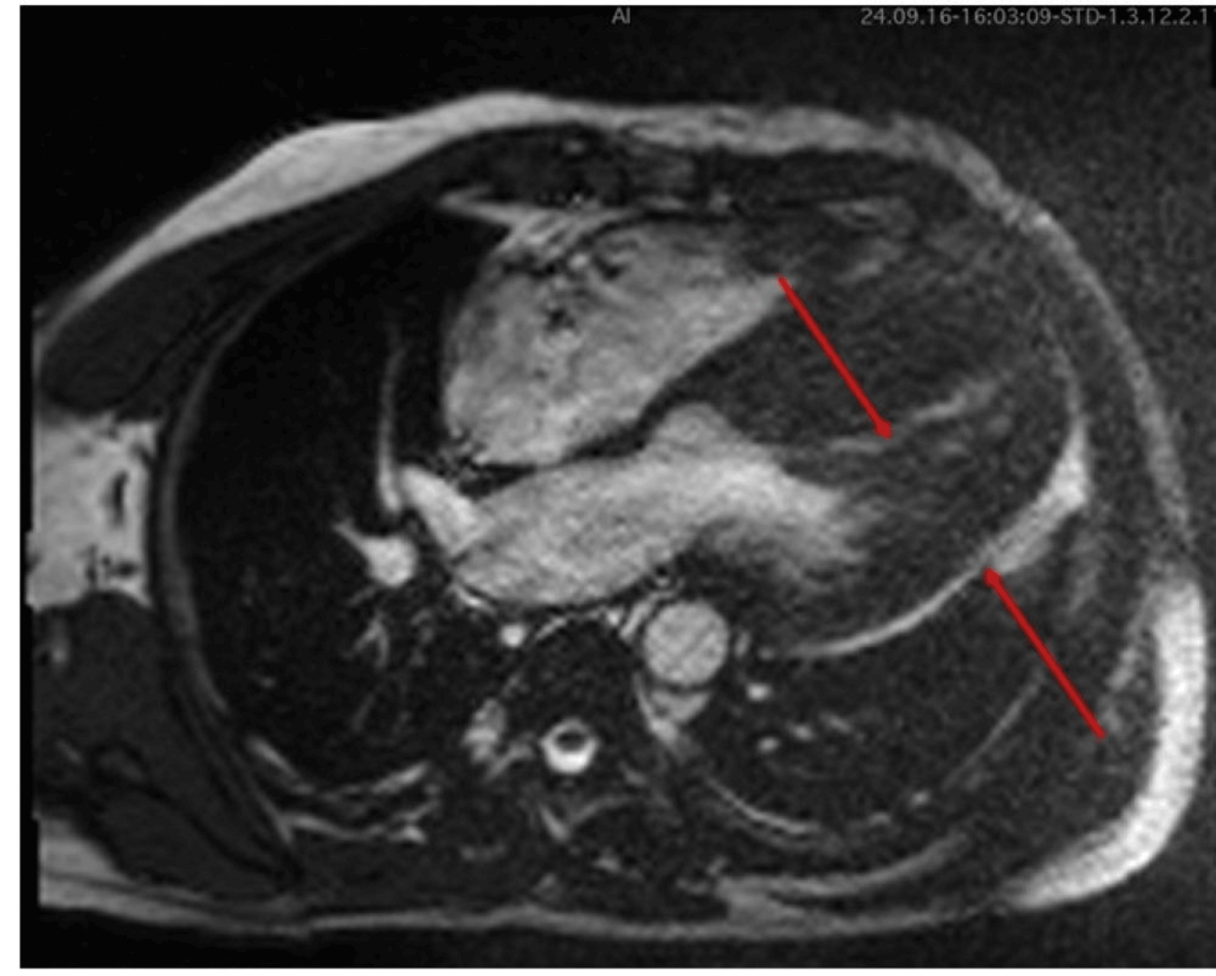
Cardiac MRI four-chamber view showing septal thickening and a small LV cavity (red arrows). LV: left ventricular.

**Figure 6:**
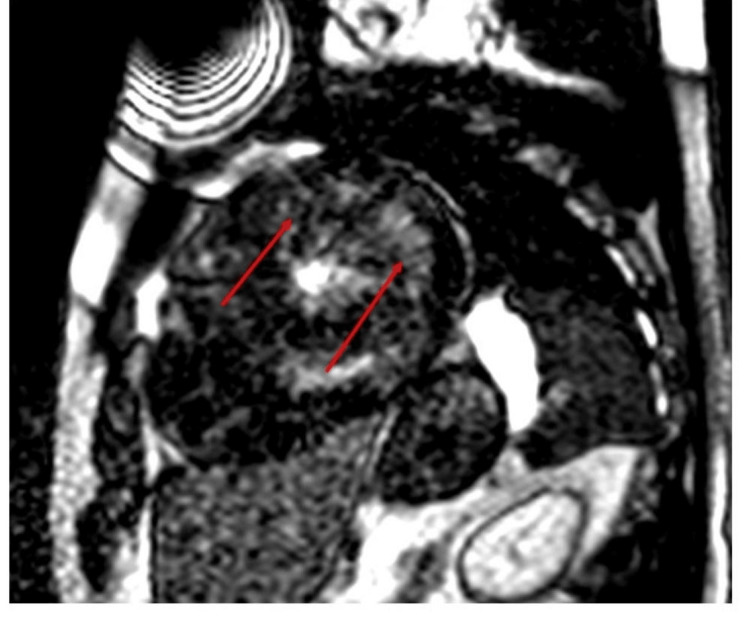
Cardiac MRI short axis showing late gadolinium enhancement (LGE) at apex (red arrows).

Based on the ECG and echocardiographic findings, a diagnosis of non-obstructive HCM and CHB was made. However, at this point, it was difficult to exclude other causes of left ventricular hypertrophy that are commonly associated with complete heart block, such as other infiltrative cardiomyopathies, but those were included on the list of possible causes. Cardiac MRI was done, which showed features of hypertrophic cardiomyopathy, with no systolic anterior mitral valve leaflet movement, an ejection fraction of 65% and late gadolinium enhancement (Figures 5, 6).

**Figure 7:**
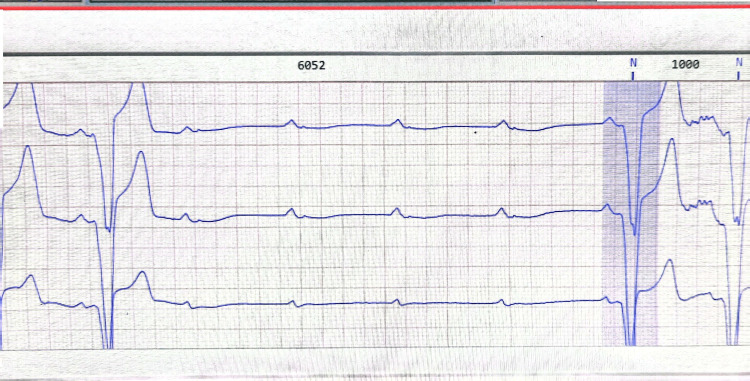
Part of the Holter strip showing significant bradycardia with a complete AV block. AV: atrioventricular.

**Figure 8:**
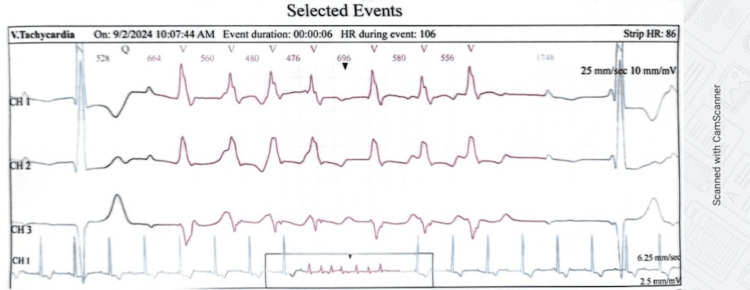
Part of the Holter strip showing a non-sustained ventricular tachycardia.

After excluding reversible causes of conduction abnormalities, such as electrolyte abnormalities, thyroid disorders, an initial temporary transvenous pacemaker was inserted due to the patient's instability. He subsequently got a dual-chamber pacemaker, rate-adaptive (DDDR), after stabilization. A 48-hour Holter (Norav Medical GmbH, Mainz-Kastel, Germany) for further risk stratification noted a complete AV block with ventricular escape rhythm (Figure 7) and two episodes of non-sustained ventricular tachycardia, with the longest of seven beats per minute, running at a rate of 145 beats per minute with isolated (Figure 8) but infrequent premature ventricular complexes with a percentage of less than one. 

The patient was discharged for review after a week. However, he returns two days later with dizziness on exertion. Physical findings were unremarkable, and he was appropriately pacing on the ECG and the monitor. Device interrogation showed no event since the implantation. The impedance in the right atrium was 546, and that of the right ventricle was 975, with all normal parameters achieved through 99% pacing. The patient was started on extended-release metoprolol succinate 25 mg, later up-titrated to 50 mg daily.

The patient got a cardiovascular magnetic resonance (CMR) evaluation, which revealed an overall systolic function of 65% with mid-wall thickness of 33 mm and no LVOT gradient. There was dense focal late gadolinium enhancement (LGE) at the RV insertion points with poorly defined late enhancement in the septum, more circumferentially towards the apex in a mid-wall pattern.

Based on the risk stratification of this patient, the mid-wall thickness of 33 millimeters noticed on the CMR evaluation, the dense focal LGE at the RV insertion points with poorly defined LGE in the septum, the two episodes of NSVT noticed on Holter monitoring, the decision was made to place a dual-chamber ICD to mitigate the risks. The patient takes metoprolol succinate 50 mg daily and is currently well, with no reported symptoms. He comes for regular outpatient visits as directed.

## Discussion

Although the association of atrioventricular block and HCM has been reported in adults, it is rare. Approximately 25% of patients with HCM experience symptoms of syncope or pre-syncope [2].

The mechanisms leading to syncope or pre-syncope can be either arrhythmic or hemodynamic in origin. Arrhythmic causes include ventricular tachycardia, which is quite common. Besides the tachyarrhythmia, bradyarrhythmia, such as atrioventricular (AV) block, can also lead to syncope, a commonly recognized phenomenon.

Conduction disturbances, such as atrioventricular (AV) block, are uncommon, and such complications are infrequent. Dubey et al. reported a 28-year-old man from Nepal who presented with syncope. ECG revealed CHB. Further evaluation revealed asymmetric septal hypertrophy. The patient eventually got a permanent pacemaker and recovered [5]. In 1977, Spilkin and associates described the case of a 20-year-old man with HCM who subsequently developed CHB. The patient had a three-year history of chest pain and dyspnea, and a physical finding of a systolic ejection murmur. Further evaluation revealed hypertrophy of the interventricular septum and systolic anterior motion of the mitral valve. Catheterization findings included a 64-mmHg subaortic gradient and left ventricular cavity obliteration. A permanent unipolar ventricular inhibited pacemaker was implanted, and his symptoms improved [6]. The last case demonstrates obstructive hypertrophic cardiomyopathy, though in our case, the patient did not have a resting or provoked LVOT gradient.

The cause of AV block in HCM is not clear. Histopathologic reports have described possible causes. A report by Kothari et al., in their review of an original work by Maron et al., described histopathologic examination of the atrioventricular nodal tissue, which was normal; however, continuity of the conduction system was interrupted in the bundle of His [7].

Bharati et al. demonstrated interstitial fibrosis or myocardial necrosis in the conduction system as well as abnormally small intramural coronary arteries with thickened walls, luminal narrowing in HCM, and advanced conduction system disorders [8]. This mechanical hypothesis of conduction disturbances is supported by animal studies. Kaneshige et al. examined histologically the cardiac conduction system of thirteen felines with HCM and CHB. Marked degeneration and fibrosis replacement of the atrioventricular conduction system were consistently observed in the combined regions of the branching portion of the atrioventricular bundle and the upper portion of the left bundle branch. These changes were associated with extensive fibrosis of the central fibrosis, endocardial and myocardial fibrosis in the upper border of the ventricular septum [9]. In another review, Fananapazir et al. performed hemodynamic and electrophysiology evaluation studies in 30 survivors of sudden cardiac arrest with HCM to determine responsible factors. Possible causes of sudden cardiac arrest were found in all patients. They were ventricular instability (70%), severe LVOT (27%), bradycardia (17%), and atrial tachycardia resulting in hypotension (13%) [10]. These reports suggest that while ventricular tachyarrhythmia is a clearly common mechanism in patients with SCD, bradyarrhythmia may also be a possible cause, and this should alert physicians in the evaluation of such patients. Notably, our patient had marked bradycardia with CHB, and while other non-invasive tests did not reveal ventricular tachycardia or a significant NSVT (beats greater than seven), it can be assumed our patient’s symptoms were likely bradyarrhythmic in nature. It can also be hypothesized that areas of fibrosis, as demonstrated by LGE, may explain this mechanism, especially if the AV node is involved.

It is also important to point out that our patient had late gadolinium enhancement (LGE) on CMR, which has a growing body of literature concerning the use of risk stratification for patients with HCM. As noted by Hoey et al., patients with LGE have been shown to have a seven-fold increased risk for potentially lethal ventricular arrhythmias compared to those without. However, the team emphasized that it is worth noting that around 65% of HCM patients will have LGE evidence of fibrosis, and LGE positivity alone may not be the best predictor of events [11]. Our patient had some LGE activity, and though not quantified, our decision for a dual-chamber ICD was based solely on the CMR mid-wall thickness of 33 millimeters, which is a significant risk factor for SCD in this population of patients.

In other reviews, however, LVOT gradient disappeared with the insertion of a dual-chamber pacemaker, as reported by Yesil et al., who described a prompt disappearance of outflow tract gradient in a 27-year-old man with obstructive hypertrophic cardiomyopathy [12]. As noted earlier, our patient did not have a resting or provoked LVOT gradient, but this is an interesting finding in the management of patients with HCM and should be noted. Another important point to note in the management of these patients is the link between genetics and some mutations that are commonly associated with conduction disturbances. Clearly, knowledge of disease-causing mutations in an index case enables rapid genetic testing and diagnosis with cascade testing in close relations.

In a review by Porto et al. on the clinical spectrum of the PRKAG2 syndrome (PS), a rare, early onset autosomal dominant inherited disease characterized by ventricular pre-excitation, supraventricular arrhythmias and cardiac hypertrophy, the team aimed to describe the various features and clinical implications of PS after reviewing a total of 193 genetically confirmed patients and 13 different mutations of PRKAG2 gene. The age of onset was seldom available. In general, the clinical onset ranged from the intrauterine period, early childhood, adolescence to the fourth or fifth decade of age. The team found 82 subjects (43%) who had permanent pacemaker implantation because of advanced heart blocks or sinus node disease [13].

This review suggests that genetic studies, which is one of the cornerstones of management, could have significant management implications and should be considered where available in patients with HCM. There have been no genetic studies performed for patients with HCM in Uganda; however, our patient would have benefited from such a study, which can detect mimickers and help initiate cascade screening in family members at risk. This is a limitation in our case review and should be noted.

## Conclusions

Our case faced management challenges, including a lack of genetic testing, which is one of the four pillars of management for such patients; however, we emphasized clinical surveillance in close relations. In addition, imaging services like CMR are not readily available to the general population, which is important in risk stratification and in equivocal situations where there are suboptimal two-dimensional echocardiography images.

Lastly, while it is common to encounter tachyarrhythmias in patients with HCM, atrioventricular block is a rare complication, and one should be aware of the possibility of it as a cause of syncope in a patient with HCM. What is more important is the recognition of the association of AV block in such patients, as it alters the approach to the treatment of each condition.
